# Enhanced Codelivery of Gefitinib and Azacitidine for Treatment of Metastatic-Resistant Lung Cancer Using Biodegradable Lipid Nanoparticles

**DOI:** 10.3390/ma16155364

**Published:** 2023-07-30

**Authors:** Ehab M. Elzayat, Abdelrahman Y. Sherif, Fahd A. Nasr, Mohamed W. Attwa, Doaa H. Alshora, Sheikh F. Ahmad, Ali S. Alqahtani

**Affiliations:** 1 Department of Pharmaceutics, College of Pharmacy, King Saud University, P.O. Box 2457, Riyadh 11451, Saudi Arabia; eelzayat1.c@ksu.edu.sa (E.M.E.); dalahora@ksu.edu.sa (D.H.A.); 2 Department of Pharmacognosy, College of Pharmacy, King Saud University, P.O. Box 2457, Riyadh 11451, Saudi Arabia; fnasr@ksu.edu.sa (F.A.N.); alalqahtani@ksu.edu.sa (A.S.A.); 3 Department of Pharmaceutical Chemistry, College of Pharmacy, King Saud University, P.O. Box 2457, Riyadh 11451, Saudi Arabia; mzeidan@ksu.edu.sa; 4 Department of Pharmacology and Toxicology, College of Pharmacy, King Saud University, P.O. Box 2457, Riyadh 11451, Saudi Arabia; fashaikh@ksu.edu.sa

**Keywords:** Gefitinib, Azacitidine, metastatic-resistant lung cancer, lymphatic delivery, NLC

## Abstract

Lung cancer is a formidable challenge in clinical practice owing to its metastatic nature and resistance to conventional treatments. The codelivery of anticancer agents offers a potential solution to overcome resistance and minimize systemic toxicity. The encapsulation of these agents within nanostructured lipid carriers (NLCs) provides a promising strategy to enhance lymphatic delivery and reduce the risk of relapse. This study aimed to develop an NLC formulation loaded with Gefitinib and Azacitidine (GEF-AZT-NLC) for the treatment of metastatic-resistant lung cancer. The physicochemical properties of the formulations were characterized, and in vitro drug release was evaluated using the dialysis bag method. The cytotoxic activity of the GEF-AZT-NLC formulations was assessed on a lung cancer cell line, and hemocompatibility was evaluated using suspended red blood cells. The prepared formulations exhibited nanoscale size (235–272 nm) and negative zeta potential values (−15 to −31 mV). In vitro study revealed that the GEF-AZT-NLC formulation retained more than 20% and 60% of GEF and AZT, respectively, at the end of the experiment. Hemocompatibility study demonstrated the safety of the formulation for therapeutic use, while cytotoxicity studies suggested that the encapsulation of both anticancer agents within NLCs could be advantageous in treating resistant cancer cells. In conclusion, the GEF-AZT-NLC formulation developed in this study holds promise as a potential therapeutic tool for treating metastatic-resistant lung cancer.

## 1. Introduction

Lung cancer is a significant global health concern, ranking as the second most prevalent cancer type worldwide [1]. The prolonged use of conventional anticancer drugs has led to a high degree of resistance against lung cancer [2]. Therefore, various approaches have emerged to overcome the developed resistance, including photothermal approaches [3], immunotherapy [4], and codelivery of chemotherapeutic agents [5]. However, the photothermal strategy has various limitations arising from the development of a strong antioxidant system within cancer cells, and difficulty in sufficient H_2_O_2_ delivery along with a low rate of free radical production [3]. On the other hand, the limitations of immunotherapy therapy result from its negative impact on the immune system as well as its limited effect on the superficial cancer cells [4]. Interestingly, the codelivery of adjunctive chemotherapeutic agents not only enhances cytotoxic activity against resistant cancer cells, but also reduces the dose of the primary drug, which decreases systemic toxicity [5].

Gefitinib (GEF) is an FDA-approved drug for the treatment of lung cancer due to its effectiveness [6]. It inhibits epidermal growth factor receptors (EGFRs), which are present on the surface of normal cells and regulate cell survival. The expression of EGFRs is triggered in various types of solid tumors, including lung cancer [7]. Numerous types of chemotherapeutic agents called tyrosine kinase inhibitors bind to the intracellular domain of EGFRs, which activate downstream protein synthesis and induce cancer cell death [8]. However, the administration of GEF can result in various systemic toxicities that necessitate reduced dosing with minimal medicinal outcomes. Moreover, the reported resistance to GEF is a prevalent issue that limits its therapeutic effect [9]. Therefore, the codelivery of anticancer agents has emerged as a hopeful approach that reduces systemic toxicity and overcomes resistance [10].

Selected adjunctive therapy with a common downstream intracellular effect and a synergetic effect achieves the required effect with maximum therapeutic outcomes [10]. Azacitidine (AZT) induces cancer cell death through increased expression of tumor-suppressor genes following the demethylation of DNA [11]. In addition, AZT has shown promising outcomes during the treatment of lung cancer [12,13]. In light of this, the codelivery of GEF and AZT could be a potential approach to overcome the acquired resistance against the former. However, conventional administration is usually associated with systemic toxicity [14]. Therefore, the encapsulation of chemotherapeutic agents within nanoparticles is required to avoid these limitations.

Nanoparticles can be tailored to deliver therapeutic agents directly to tumor cells based on their inherent permeability through large fenestrations present on blood vessels around the tumor mass [15], which limits the exposure of healthy organs and tissues to anticancer agents and reduces systemic toxicities [14]. Therefore, various types of nanocarriers have been prepared from polymers [16], metals [17], inorganic materials [18], and lipid nanoparticles [8] to achieve this purpose. However, encapsulating anticancer agents within a drug delivery system capable of reaching the lymphatic system and overcoming resistance offers a promising approach to improve patient outcomes. This is attributed to the reported cancer relapse, where detached cancer cells are hidden within the lymph nodes of the lymphatic system [19] and can enter systemic circulation following therapeutic protocols, leading to the formation of new masses in systemic organs [20].

Tailoring nanoparticles from lipid materials offers an additional advantage in therapeutic outcomes. It is well-established that lipid nanoparticles possess innate distribution to the lymphatic system, which enhances the complete elimination of hidden cancer cells [19]. Additionally, various types of lipid receptors are overexpressed on the surface of cancer cells, which potentiates the active delivery of anticancer-loaded agents [21,22]. To further enhance the advantages of selected lipids, stearic acid and oleic acid were chosen due to their ability to encapsulate weak base drugs containing amino groups [23]. Interestingly, nanoparticles larger than 200 nm are susceptible to lung accumulation due to reinforced infiltration and sequestration by pulmonary small capillaries [24].

In this study, the potential therapeutic outcomes of co-delivering GEF and AZT were investigated following the preparation of various nanostructured lipid carrier (NLC) formulations. The formulations were subjected to physicochemical characterization, including size, zeta potential, and morphology analyses. Furthermore, the prepared formulations were evaluated for in vitro drug release, cytotoxicity, and hemocompatibility studies.

## 2. Materials and Methods

### 2.1. Materials

Gefitinib was purchased from Beijing Mesochem Technology Co., Ltd. (Beijing, China). Azacitidine was kindly obtained from Sudairpharma Company (Riyadh, Saudi Arabia). Poloxamer-188 (P-188) and D-α-Tocopherol polyethylene glycol 1000 succinate (TPGS) were purchased from Sigma Aldrich, St. Louis, MO, USA. Stearic acid (SA) was purchased from BDH (Poole, UK). All other chemicals and reagents were of pharmaceutical or analytical grade.

### 2.2. Analysis of Gefitinib and Azacitidine in Formulations

**Figure 1:**
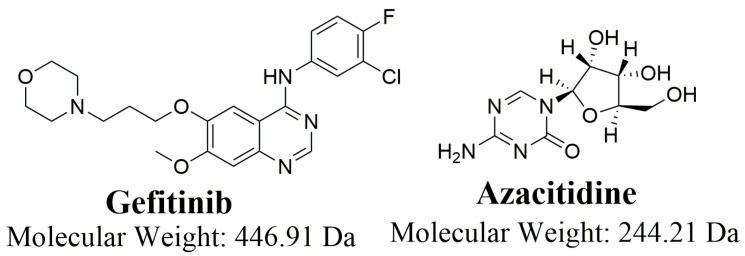
Chemical structure of Gefitinib and Azacitidine.

In this study, a validated UPLC-MS/MS (UPLC: Waters Acquity, Milford, MA, USA) was employed to determine the concentration of Gefitinib and Azacitidine (Figure 1) in tablet formulation. The chromatographic conditions involved the use of a Zorbax Eclipse Pllus C18 column (50 mm × 2.1 mm, 1.8 µm, Agilent Technologies, Santa Clara, CA, USA) with a mobile phase of acetonitrile and 0.1% formic acid (50: 50 *v*/*v*) in an isocratic elution running at a flow rate of 0.2 mL/min for a total run time of 3 min. The eluted compounds were detected with tandem mass spectrometry using TQ detector (Waters Corp., Milford, MA, USA) equipped with an electrospray ionization (ESI) source operating in positive ionization mode. The instrumentation parameters are displayed in Table 1.

### 2.3. Preparation of NLC Formulations

NLC formulations were prepared using the ultrasonic melt-emulsification process, as previously reported [25]. The actual composition of ingredients used in the preparation of NLC formulations is shown in Table 2. The aqueous phase was prepared by dissolving 120 mg of Poloxamer-188 (P-188) and 7.5 mg of D-α-Tocopherol polyethylene glycol 1000 succinate (TPGS) in water contained in a cylindrical beaker. The lipid phase was prepared by adding accurately weighed stearic and oleic acid in another cylindrical beaker. GEF, AZT, and GEF + AZT were added to the lipid phase to prepare GEF-NLC, AZT-NLC, and GEF-AZT-NLC, respectively. Both aqueous and lipid phases were heated simultaneously at 80 °C. The hot aqueous phase was gradually added to the hot lipid phase and stirred for 1 min to obtain a primary emulsion. The primary emulsion was then subjected to nine cycles of ultrasonication, each one extending for 20 s, followed by a resting period extending for 10 s. Immediately after preparation, NLC formulations were kept in the freezer for 10 min to facilitate lipid solidification. Directly after cooling, the NLC formulation was subjected to centrifugation at 3000 rpm for 3 min to precipitate insoluble particles. The upper portion of the centrifuged formulation was transferred into a new vial and kept in the refrigerator for further investigation.

### 2.4. Physicochemical Characterization

#### 2.4.1. Particle Size, Polydispersity Index, and Zeta Potential Measurements

The physicochemical characteristics of the prepared formulations were assessed utilizing a Zetasizer Nano ZS (Malvern Instruments, Malvern, UK). Each formulation was assessed at 25 °C after being diluted in distilled water (1:10,000). Particle size, polydispersity index, and zeta potential were measured using the Dynamic Light Scattering (DLS) and Laser Doppler Velocimetry (LDV) modes. Each value was represented as the average across three separate repetitions [26].

#### 2.4.2. Calculation of Drug Content and Entrapment Efficiency

Drug content was measured to calculate the amount of drug present in a particular volume of the formulation. Briefly, 1 mL of the formulation was placed in a 25 mL volumetric flask and diluted with methanol up to the final volume. It was directly subjected to sonication for 15 min to dissolve NLC and ensure complete drug extraction from the formulation. The resulting solution was diluted (1:10) in acetonitrile, and drug concentration was determined using the developed UPLC method [9]. The following equation was utilized to calculate the drug content:

$$\mathrm{drug}\;\mathrm{content}=\frac{\mathrm{The}\;\mathrm{total}\;\mathrm{amount}\;\mathrm{of}\;\mathrm{drug}\;\left(\mathrm{mg}\right)}{\mathrm{Volume}\;\mathrm{of}\;\mathrm{NLC}\;\mathrm{formulation}\;\left(\mathrm{mL}\right)}\;\times \;100$$

The percentage of GEF and AZT entrapped within the prepared NLC formulation was determined using an indirect method [27]. The freshly prepared formulation was subjected to centrifugation at 50,000 rpm for 30 min to precipitate NLC formulation. The supernatant was filtered to remove any possible residue from NLC formulation and drug concentration was measured using the developed UPLC method. The following equation was utilized to calculate the entrapment efficiency:

$$\begin{array}{c} \mathrm{Entrapment}\;\mathrm{efficiency}\\ =\frac{\mathrm{The}\;\mathrm{total}\;\mathrm{amount}\;\mathrm{of}\;\mathrm{drug}\;\left(\mathrm{mg}\right)-\;\mathrm{The}\;\mathrm{amount}\;\mathrm{of}\;\mathrm{drug}\;\mathrm{in}\;\mathrm{the}\;\mathrm{supernatant}\;\left(\mathrm{mg}\right)}{\mathrm{The}\;\mathrm{total}\;\mathrm{amount}\;\mathrm{of}\;\mathrm{drug}\;\left(\mathrm{mg}\right)}\;\times \;100 \end{array}$$

### 2.5. Differential Scanning Calorimetry (DSC)

Thermal analysis was performed to scan thermal behavior of GEF, AZT, stearic acid, P-188, plain-NLC, AZT-NLC, GEF-NLC, and GEF-AZT-NLC using a DSC 8000 Perkins Elmer (Waltham, MA, USA) apparatus in the temperature range of 25–250 °C at a heating rate of 10 °C/min. Pyris management software version 10.1 (Pyris Elmer, Waltham, MA, USA) was utilized for the solid-state characterization and assessment of the samples [28].

### 2.6. Powder X-ray Diffractometer (PXRD)

PXRD was used to investigate the crystalline state of GEF and AZT within the prepared NLC formulation. This was attained via the detection spectrum of GEF, AZT, stearic acid, plain-NLC, AZT-NLC, GEF-NLC, and GEF-AZT-NLC. This was performed at a 0.5/min scanning rate within the theta scanning range 3–180° [26].

### 2.7. In Vitro Release Study

The dialysis bag method was utilized to evaluate GEF and AZT release from the prepared formulations [23,29]. About 0.5 mL from each formulation was diluted (1:4) and placed within a dialysis membrane bag (molecular-weight cut-off: 12–14 kDa). The sealed bag was placed in a previously heated beaker containing 50 mL of phosphate buffer (pH 7.4, with 0.5% Tween-80, Loba Chemical Company, Bombay, India). The beaker was continuously shaken at 100 rpm at 37 ± 1 °C in a thermostat shaker. Samples were withdrawn at 0.25, 0.50, 1.0, 2.0, 4.0, 8.0, 12.0, and 24.0 h and an equal amount of dissolution media was replaced. The withdrawn samples were centrifuged for 10 min at 10,000 rpm and the amount of drug in the supernatant was determined using the developed UPLC method. It should be noted that the effect of the membrane was previously studied in published data [26].

### 2.8. In Vitro Cytotoxicity Study

As a lung cancer model, the Human non-small-cell lung cell line (A549) was obtained from DSMZ Leibniz Institute (German Collection of Microorganisms and Cell Cultures Braunschweig, Germany) and used to examine the cytotoxicity of the prepared NLC formulations. Cells were grown in DMEM culture media (Gibco, Thermo Fisher Scientific, Waltham, MA, USA) and kept at 37 °C in an incubator. Furthermore, 1% *v*/*v* penicillin-streptomycin (Gibco, Thermo Fisher Scientific, Waltham, MA, USA) and 10% *v*/*v* FBS (Gibco, Thermo Fisher Scientific, Waltham, MA, USA) with 5% CO_2_ were added to the cells during incubation. Then, a 96-well plate with 1 × 10^5^ cells per well was allowed to develop for 24 h. After treatment with NLC formulations at four different doses (2.5–10 μg/mL), the growing cells were incubated for 24 h. After that, each well was treated with 10 μL of the MTT solution (5 μg/ mL, Invitrogen, Waltham, MA, USA) before being placed in an incubator at 37 °C for 4 h in the dark. After being dissolved in acidified isopropanol (Sigma-Aldrich, St. Louis, MO, USA), the formazan product’s absorbance was determined using a microplate reader at 570 nm. The concentration needed to prevent cell growth by 50% (IC_50_) was calculated via a dose–response curve. Cell viability was calculated according to the following equation [30].

Cell Viability (%) = (optical density of the treated sample)/(optical density of the untreated sample) × 100%.

### 2.9. Hemocompatibility Study

To evaluate the hemocompatibility of the GEF-AZT-NLC formulation, a blood sample (Experimental Animal Care Center, King Saud University, Riyadh, Saudi Arabia) was utilized [9]. To create a 2% erythrocyte suspension, red blood cells were diluted with a physiological saline solution (Pharmaceutical Solutions Industry, Jeddah, Saudi Arabia). The GEF-AZT-NLC formulation was incubated with the suspended red blood cells at various concentrations (2.5, 5.0, and 10.0 g/mL) equivalent to the cytotoxicity study. To prepare the negative and positive controls, erythrocyte suspension was treated with physiological saline solution and ultra-pure water, respectively. The suspended red blood cells were vortexed and maintained in the incubator at 37 °C for one hour. The incubated erythrocyte cells were centrifuged at 14,000 rpm for 10 min directly after incubation. Each formulation’s concentration was evaluated three times. We used a UV-Vis spectrophotometer to calculate the absorbance at 570 nm. The following equation was utilized to estimate the hemolysis ratio.

$$\mathrm{Hemolysis}=\frac{\mathrm{Absorbance}\;\mathrm{sample}-\mathrm{absorbance}\;\mathrm{negative}\;\mathrm{control}}{\mathrm{absorbance}\;\mathrm{positive}\;\mathrm{control}-\mathrm{absorbance}\;\mathrm{negative}\;\mathrm{control}}\times 100$$

### 2.10. Statistical Analysis

All data analyses were performed using SPSS software, v26. The results were compared using a *t*-test and a one-way analysis of variance (ANOVA) followed by Tukey’s test to compare three or more data sets. Data were expressed as mean ± SD. *p*-value < 0.05 was used as the criterion for significance.

## 3. Results and Discussion

### 3.1. Method Performance and Assay Validation

**Scheme 1:**
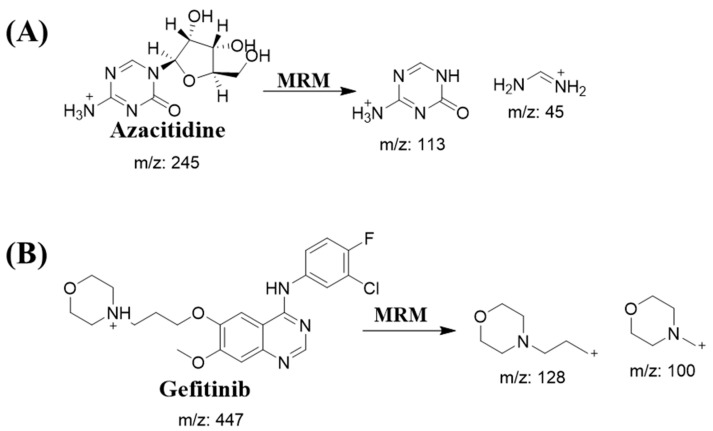
MRM mass transitions for (**A**) Azacitidine and (**B**) Gefitinib.

**Figure 2:**
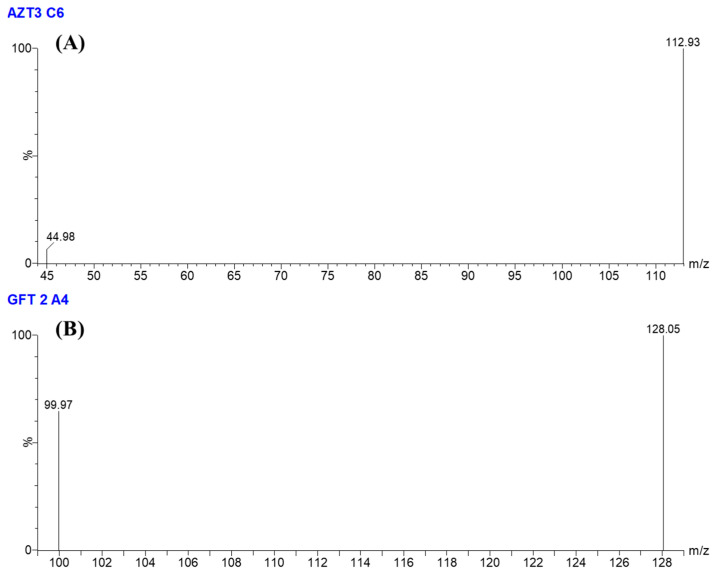
MRM mass spectrum of (**A**) Azacitidine and (**B**) Gefitinib.

The quantification was performed with multiple reaction monitoring (MRM) mode. The selection of ionization pairs (*m*/*z*) was shown as follows: Gefitinib—447.12 → 128.06 (cone voltage 40 V, collision energy 26 V) and 447.12 → 99.97 (cone voltage 40 V, collision energy 52 V), Azacitidine—245.05 → 112.93 (cone voltage 16 V, collision energy 8 V) and 245.05 → 112.93 (cone voltage 16 V, collision energy 8 V). MRM mass transitions are displayed in Scheme 1 and Figure 2.

**Figure 3:**
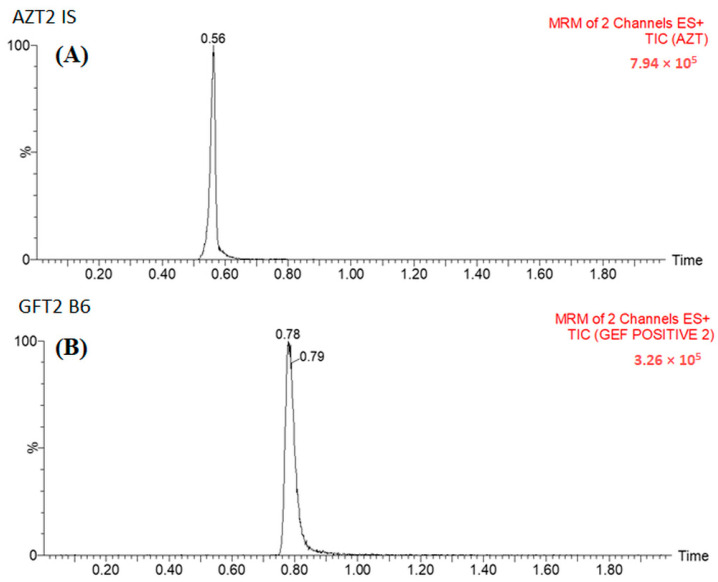
MRM chromatogram of (**A**) Azacitidine and (**B**) Gefitinib.

Several combinations of acetonitrile and 0.1% formic acid were evaluated as possible mobile phases. It was determined that the combination of acetonitrile and 0.1% formic acid in an isocratic elution program (50: 50 *v*/*v*) was found to be the most suitable for separating Gefitinib and Azacitidine, as shown in Figure 3. Under the described chromatographic conditions, the retention time was about 0.56 and 0.78 min for Azacitidine and Gefitinib, respectively (Figure 3).

Good linearity (r^2^ > 0.997) was observed over the range of 0.1–5 µg/mL and for Gefitinib and Azacitidine and could be described by the regression equations: Y = 247 X − 24,431 (Gefitinib) and Y = 388.18 X − 38,392 (Azacitidine), in which Y is the peak area of the drug and X is the analyte concentration in µg/mL in the samples.

### 3.2. Physicochemical Properties of Prepared NLCs

Table 3 displays the physicochemical properties of the successfully prepared NLC formulations. The prepared formulations were found to be in the nano-size range with a high degree of homogeneity, as indicated by a polydispersity index value of ≤0.2. It is clear from the results that the particle sizes of all formulations were almost the same. Interestingly, the prepared formulations were in the nano-size range of 200 to 300 nm, indicating their suitability for lung drug delivery [24]. The zeta potential value of the prepared plain-NLC was negatively charged and measured to be less than −30 mV, which can be attributed to the incorporation of stearic acid, in agreement with published studies [28,31]. The addition of GEF and AZT was found to increase the zeta potential value, which could be attributed to the neutralization effect produced by the free amino group of GEF and AZT [26,32]. Furthermore, the neutralization effect was predominant in the case of AZT-NLC. This could be attributed to the partial presence of AZT on the surface of nanoparticles owing to its hydrophilicity [33].

The prepared formulations showed a drug content ranging from 0.98 to 1.04 mg/mL with an incredible entrapment efficiency of above 95% for both drugs. This can be attributed to the selection of long-chain fatty acids that were able to solubilize both drugs within the lipid core of the NLC formulation [23]. The obtained results are in harmony with previously published data by Fernandes et al., who prepared NLCs consisting of oleic acid loaded with a hydrophilic drug with a free amino group. The optimized formulation showed remarkable entrapment efficiency (97%), and the author attributed this to the ion-pair formation between the free carboxylic acid of oil and the free amino group of the drug [34]. Moreover, various studies showed a positive correlation between the solubility of loaded hydrophilic drugs in different types of lipids and the obtained entrapment efficiency [35,36]. The impact of the concentration of lipids on the entrapment efficiency of a hydrophilic drug was reported by Yanga et al. It was found that doubling the concentration of the lipid and drug, while keeping the concentration of the aqueous phase constant, resulted in increasing the entrapment efficiency from 86 to 97% [37]. In the present study, the incredible entrapment efficiency could be attributed to the selection of lipids with high drug solubility at high concentrations (5%).

### 3.3. DSC

**Figure 4:**
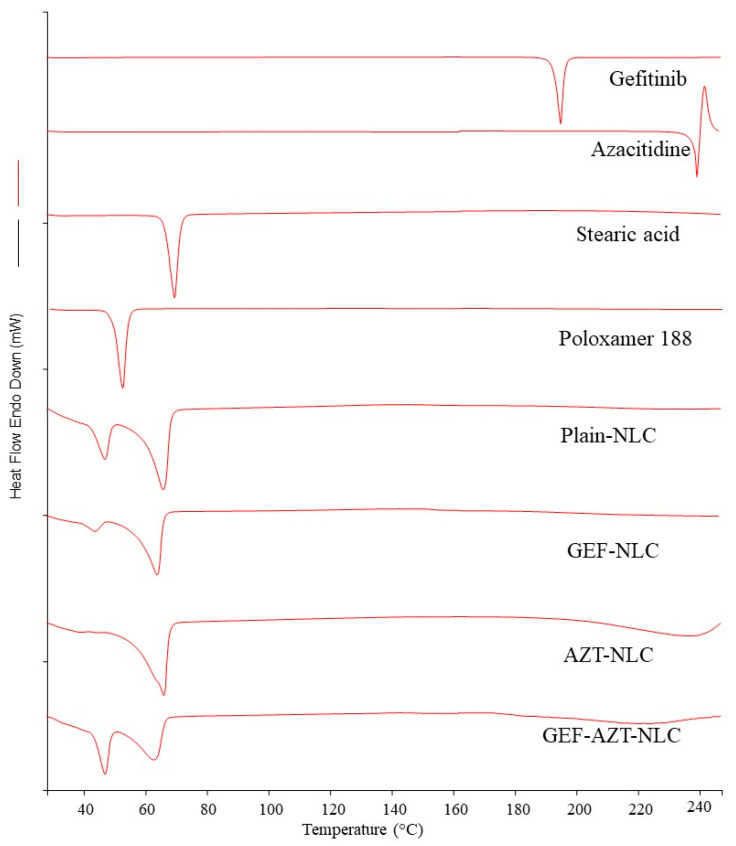
DSC of Gefitinib, Azacitidine, stearic acid, poloxamer 188, plain-NLC, GEF-NLC, AZT-NLC, and GEF-AZT-NLC.

Figure 4 displays the DSC profiles of the pure APIs, pure excipients, and the prepared NLC formulations. The curves showed an endothermic peak of the pure GEF and AZT at 196 and 240 °C, respectively, which is consistent with previously reported studies [26,38]. Additionally, the observed melting point of stearic acid and poloxamer-188 were at 71.0 and 53.8 °C, respectively. All NLC formulations displayed two endothermic peaks, which could represent stearic acid and poloxamer 188. The observed right shift and broadening in peaks compared with pure excipients can be attributed to the presence of oleic acid [28,39]. The melting peaks of GEF and AZT disappeared in all loaded formulations owing to the dilution effect produced by the formulation or the conversion of the APIs from a crystalline to the amorphous form [38,40]. Thus, the prepared formulations were subjected to PXRD analysis for more investigation.

### 3.4. PXRD

**Figure 5:**
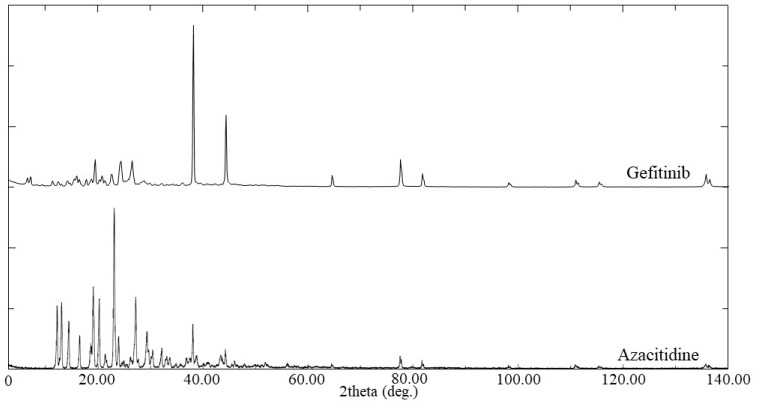
PXRD of Gefitinib and Azacitidine.

X-rays are commonly used to investigate the crystalline state of APIs and excipients in prepared nanoparticle formulations. However, it was necessary to study the crystalline state of pure APIs and excipients initially to compare the PXRD spectrum with that of the NLC formulations. Figure 5 illustrates the PXRD spectrum of pure GEF and AZT. The PXRD spectrum of GEF displayed two predominant peaks at 38.1 and 44.3 °C, in addition to moderate peaks at 19.4 and 77.5 °C, which is consistent with the reported spectrum by Alshehri et al. [41]. The spectrum shows that AZT had multiple peaks between 12.2 and 29.3 °C, with an additional peak at 38.0 °C, in agreement with previously reported studies by Kesharwani et al. [29].

**Figure 6:**
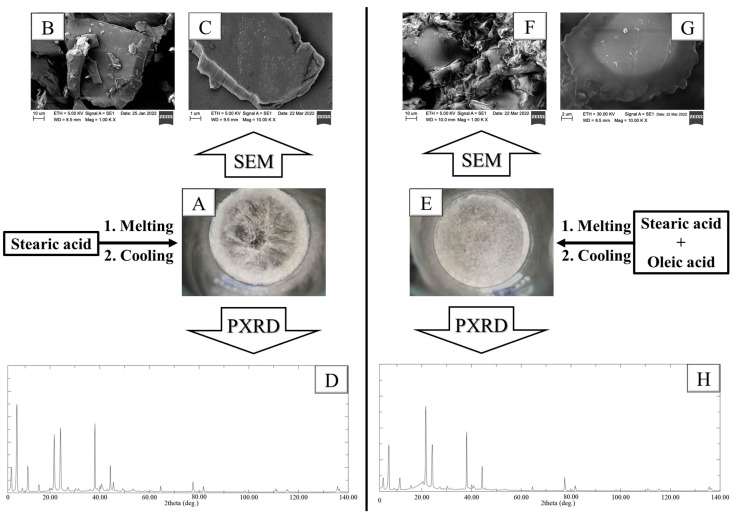
(**A**) Morphological appearance, (**B**,**C**) crushed crystal SEM images with low and high magnification power, (**D**) crushed crystal PXRD pattern of stearic acid. (**E**) Morphological appearance, (**F**,**G**) crushed crystal SEM images with low and high magnification power, (**H**) crushed crystal PXRD pattern of stearic acid and oleic acid. The lipid (s) was melted and mixed to acquire homogenous mixture, then left to cool.

Observing the crystalline state of the nanoparticle core with SEM is challenging. Therefore, the lipids used (oleic and stearic acids) were mixed in a 10 mL cylindrical beaker and heated to melt the lipids, then left to cool. Additionally, stearic acid alone underwent a similar process to observe its normal crystalline state without oleic acid. Figure 6A,E demonstrate that stearic acid and mixed lipids (oleic and stearic acids) had a crystalline arrangement, with the former showing a more pronounced effect. Furthermore, the processed lipids were crushed in a mortar and subjected to SEM to precisely observe the crystalline state. Figure 6B,C display an image of crushed processed stearic acid with low and high magnification power, respectively. The low magnification power (Figure 6B) showed that stearic acid has a highly organized crystalline structure following heating and cooling. Moreover, Figure 6C revealed that the surface of processed stearic acid is solid and impactful.

Crushes of mixed lipids (oleic and stearic acids) are also shown in Figure 6F,G, with low and high magnification power, respectively. The addition of oleic acid resulted in a significant reduction in stearic acid crystallinity. The crushes underwent PXRD examination to observe the impact of oleic acid on the crystalline pattern of stearic acid. Figure 6D shows that stearic acid had multiple peaks at 6.7, 21.7, 24.4, and 38.1 °C, with high intensity, in addition to multiple peaks at 11.1 and 44.3 °C, with moderate intensity. It is evident from Figure 6H that the addition of oleic acid during the processing of stearic acid resulted in a reduction in peak intensities. This reduction could be attributed to the liquid state of oleic acid, which prevents proper packing of stearic acid molecules during the cooling process. This was confirmed with DSC, which demonstrated a right shift and broadening in the endothermic peak of the lipid mixture (oleic and stearic acids) compared with stearic acid alone. These findings are consistent with previously reported studies, which indicated that the addition of liquid oil to a solid lipid results in decreased lipid crystallinity [42,43].

**Figure 7:**
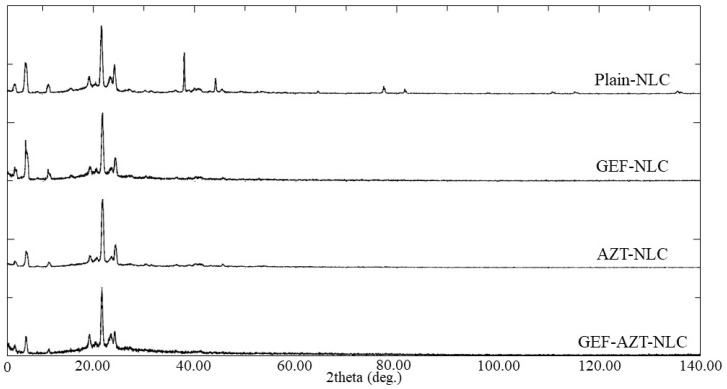
PXRD of plain-NLC, GEF-NLC, AZT-NLC, and GEF-AZT-NLC formulations.

To examine the impact of loaded drugs on the crystalline state of the lipid core, the prepared NLC formulations underwent PXRD scanning (Figure 7). The PXRD spectrum of plain-NLC displayed multiple peaks at two theta degrees, similar to the crushes obtained from the lipid mixture (oleic and stearic acids), indicating the reliability of this method to predict the crystalline lipid core of nanoparticles. It was discovered that the incorporation of GEF and AZT within the prepared NLCs resulted in the disappearance of peaks at 38.0, 44.2, and 77.6°, along with a reduction in peak intensity at 6.7°. This reduction could be attributed to the disruption of the lipid core crystallinity produced by the encapsulated drug(s), which is consistent with previously reported studies [44,45]. Furthermore, this indicates the successful loading of the incorporated drug during preparation within the NLC formulations.

### 3.5. In Vitro Release Study

The conventional administration of anticancer drugs is often associated with systemic toxicity and minimal therapeutic outcomes [14]. Therefore, drug-loaded nanoparticles have emerged as a means to enhance drug distribution to tumor tissues, decreasing systemic side effects and increasing therapeutic outcomes [46]. Drug-loaded nanoparticles are distributed to tissues through the large fenestrations present in the blood vessels of tumor tissues [47]. However, nanoparticles that can retain drugs until they reach the tumor mass are required. In light of this, an in vitro release study was conducted to predict drug release from the prepared NLC formulations in vivo.

**Figure 8:**
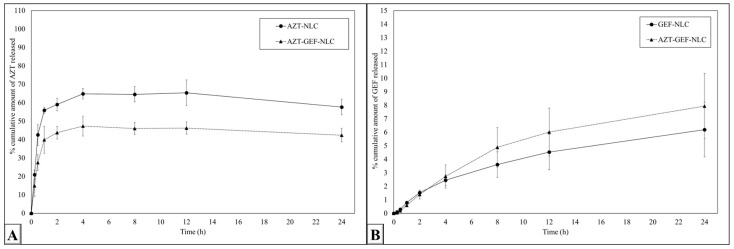
In vitro (**A**) AZT and (**B**) GEF release from the prepared NLC formulations. Data are expressed as the mean ± SD, N = 3.

Figure 8A,B display the in vitro release profile of AZT and GEF from the prepared NLC formulations. It is evident from Figure 8B that GEF was sustainably released from the GEF-NLC and GEF-AZT-NLC formulations. This could be attributed to the encapsulation of GEF within the lipid core and its low solubility. These results are consistent with previously reported studies [40,48]. Figure 8A shows that AZT release from the AZT-NLC and GEF-AZT-NLC formulations exhibited burst drug release followed by sustained drug release. The burst drug release could be attributed to the notable amount of drug present on the surface of the prepared NLC formulation due to its hydrophilic nature. On the other hand, sustained drug release could be attributed to the acidic microenvironment created by free fatty acids, facilitating the ionization and retention of AZT. These results are in agreement with previously reported studies that showed burst drug release from NLCs loaded with hydrophilic drugs. Moreover, the in vitro release study showed the ability of a loaded system with ion-pair phenomena to retain loaded hydrophilic drugs [36,49]. Additionally, Kesharwani et al. and Kashyap et al. found that AZT was released faster from nanoparticles in slightly acidic media compared with neutralized media [29,50]. This is expected to be beneficial in vivo, whereas loaded drugs could be released in acidic tumor microenvironments based on previous reported studies [51]. This will reduce systemic toxicity compared with the conventional administration of chemotherapeutic agents [19], which is in harmony with previous published data [52,53,54]. However, future in vivo studies are required to examine the impact of NLCs on drug distribution and determine the optimum drug loading and administrated doses.

### 3.6. In Vitro Cytotoxicity against Lung Cancer Cell Line

**Figure 9:**
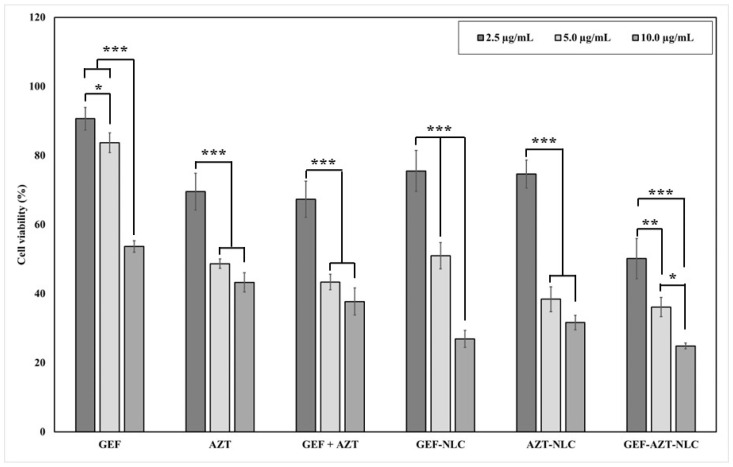
Effect of GFT, SIM, hybrid GFT + SIM, and GFT/SIM-NLC on A549 cell viability following 24 h incubation time. Data are expressed as mean ± SD, N = 3, *p*-value significant at * 0.05, ** 0.01, and *** 0.001.

In this study, the MTT assay was utilized for two purposes. The first purpose was to investigate and compare the benefits of co-treating lung cancer cells with GEF and AZT compared with each drug alone. Therefore, three groups of cell lines were treated with GEF alone, AZT alone, and GEF + AZT. The second purpose was to investigate the impact of the prepared NLC formulations on the cellular uptake of anticancer agents. Thus, an additional three groups were treated with the prepared NLC formulations (GEF-NLC, AZT-NLC, and GEF-AZT-NLC). Finally, the A549 cell line was treated with the plain-NLC formulation; an equivalent volume from each formulation corresponding to the drug concentration was incubated to ensure the safety of the prepared formulation. Figure 9 displays the cell viability results of the lung cancer cell line treated with the aforementioned seven groups. It was discovered that all tested groups exhibited a concentration-dependent cytotoxic effect after 24 h of incubation time.

#### 3.6.1. Effect of Anticancer Agents’ Codelivery

**Figure 10:**
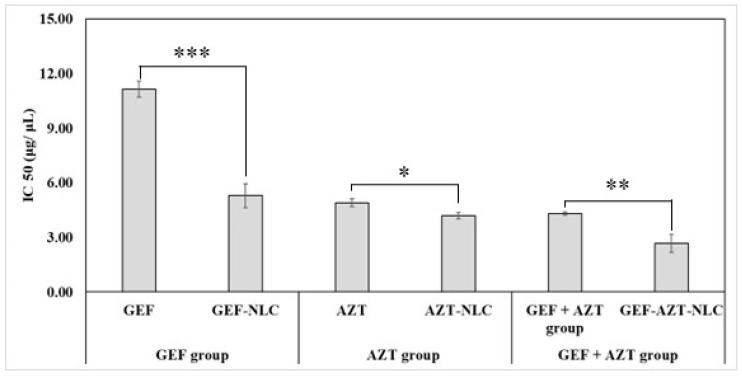
IC_50_ of GEF, GEF-NLC, AZT, AZT-NLC, GEF + AZT, and GEF-AZT-NLC following 24 h incubation time with lung cancer cell line. Data are expressed as mean ± SD, N = 3, *p*-value significant at * 0.05, ** 0.01, and *** 0.001.

To investigate the potential benefits of co-delivering GEF and AZT, a lung cancer cell line was treated with GEF alone, AZT alone, and GEF + AZT. The cell viability results (Figure 10) indicate that GEF exhibits low cytotoxic activity at lower concentrations (2.5 and 5 μg/mL), which could be attributed to its reported higher susceptibility to efflux transporters [55]. Conversely, GEF exhibits significantly extreme cytotoxic activity at high concentrations (10 μg/mL), which could be attributed to its abundance and surpassing the capacity of efflux transporters. This finding is consistent with the previously reported development of lung cancer resistance against GEF [56,57]. Therefore, there is a need for adjunctive anticancer molecules to overcome this resistance. It was found that AZT had incredible cytotoxic activity at the lowest concentration (2.5 μg/mL) that significantly increased at higher concentrations (5 and 10 μg/mL). Moreover, it was observed that the AZT-treated group exhibited a lower IC_50_ value compared with the GEF-treated group. Likewise, codelivery of both agents resulted in notable cytotoxic activity at the lowest concentration (2.5 μg/mL) which significantly increased at higher concentrations (5 and 10 μg/mL). Furthermore, co-delivering GEF + AZT resulted in a reduction in the IC_50_ value (4.31 ± 0.10 μg/mL) compared with each drug alone (Table 4). This suggests that the proposed codelivery approach could be beneficial for use in a clinical setting.

#### 3.6.2. Effect of NLC Formulations

It is clear from the results that the prepared formulation was safe and that the IC_50_ value cannot be calculated, which is in aliment with previous studies [58,59]. Figure 10 displays the IC_50_ value after 24 h of incubation of GEF, GEF-NLC, AZT, AZT-NLC, GEF + AZT, and GEF-AZT-NLC. It was discovered that incorporating the anticancer agents within the prepared NLC formulation significantly reduced the IC_50_ value compared with the free drugs. Specifically, GEF-NLC, AZT-NLC, and GEF-AZT-NLC reduced the IC_50_ value by 2.11-, 1.17-, and 1.61-fold, respectively, compared with the corresponding free drugs. Even though the NLCs had a certain dose-dependent decrease in cell viability, the observed enhancement in the cytotoxic activity of drug-loaded NLC groups could be attributed to the following reasons. At the lowest concentration (2.5 μg/mL), there was a significant difference between the cell viability of both groups (GEF + AZT and GEF-AZT-NLC). This could be attributed to the susceptibility of free chemotherapeutic agents to the efflux transporter, whereas the group treated with GEF-AZT-NLC showed enhancement in cell death activity [26]. These results are consistent with previous studies that have reported enhanced cellular uptake of anticancer agents when incorporated within nanoparticles [8,40]. Additionally, lipid nanoparticles have a high affinity for active delivery through fatty acid-binding protein receptors that are overexpressed on the surface of cancer cells [21,22]. Following cellular uptake, the presence of Poloxamer-188 and TPGS prevents drug efflux to the extracellular space through their reported efflux inhibitory activity [60,61]. On the contrary, at high concentrations (5.0 and 10.0 μg/mL), the absence of difference could be attributed to the reported overcoming of the capacity of efflux transporters’ capacity [26].

### 3.7. Hemocompatibility Study

**Figure 11:**
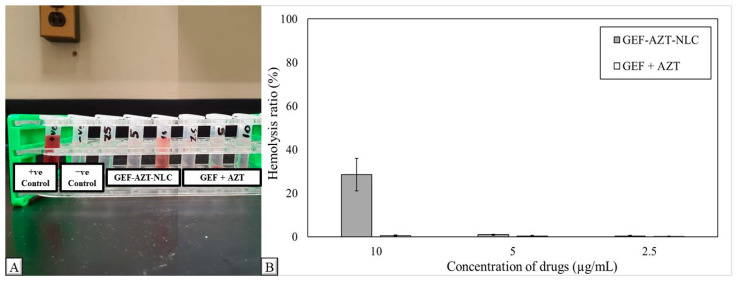
(**A**) Morphological appearance and (**B**) histogram of hemolysis ratio obtained from suspended red blood cells incubated with GEF-AZT-NLC formulation at three different concentrations (2.5, 5.0, and 10.0 µg/mL) along with positive and negative control. Data are expressed as the mean ± SD, N = 3.

The current study aimed to investigate the influence of the prepared GEF-AZT-NLC formulation on red blood cells. Red blood cells were also incubated with free drugs (GEF + AZT) to compare their effects with those of the drugs loaded in the NLC formulation. Figure 11A displays the effects of the GEF-AZT-NLC formulation and the GEF + AZT solution on the integrity of red blood cells at three different concentrations (2.5, 5.0, and 10.0 µg/mL). Figure 11B shows the histogram of the hemolysis ratio of red blood cells following incubation with the GEF-AZT-NLC formulation and GEF + AZT solution. As shown in the figure, all red blood cells were completely ruptured following incubation with hypotonic water, which represented the positive control. Conversely, the integrity of red blood cells was retained following incubation with physiological saline solution, which indicates its validity as a diluent with no influence on cell integrity. The findings demonstrate that the GEF + AZT solution has no influence on the integrity of red blood cells. Similar observations were made with red blood cells incubated with the GEF-AZT-NLC formulation at lower concentrations (2.5 and 5.0 µg/mL). However, the hemolysis ratio increased to 28% following incubation with the GEF-AZT-NLC formulation at a higher concentration (10.0 µg/mL).

The results suggest that the GEF-AZT-NLC formulation may be acceptable at concentrations lower than 5 µg/mL. The observed hemolysis at the high concentration (10.0 µg/mL) could be attributed to the presence of oleic acid. This could be linked to its liquid nature and negative charge, which alters the surface properties of red blood cells (41). The current findings are consistent with previous studies, which have revealed a connection between red blood cell hemolysis and the inclusion of free fatty acids [62,63,64]. Our previous study showed that conventional administration of GEF at the recommended dose produces a serum level below 2 µg/mL. Additionally, normal blood samples were collected following the administration of the NLC formulation, and the rats survived until the end of the experiment [28]. Therefore, the prepared GEF-AZT-NLC formulation could be acceptable after in vivo administration.

## 4. Conclusions

The present study focused on preparing the GEF-AZT-NLC formulation in the nano-size range, which holds promise for treating metastatic-resistant lung cancer. The prepared formulation was able to encapsulate both drugs with incredible encapsulation efficiency, and in vitro release studies revealed that the NLC was able to retain the drugs. In vitro cytotoxicity studies demonstrated that the codelivery of AZT with GEF significantly enhanced its cytotoxic activity. Moreover, the encapsulation of both drugs in the NLC was able to enhance their internalization and cytotoxic activity. However, additional in vivo studies and clinical trials are still required to confirm the current achievements in animals and humans.

## Figures and Tables

**Table 1:** Analytical parameters of liquid chromatography and mass detection.

Acquity UPLC (H10UPH)		Acquity TQD MS (QBB1203)	
Isocratic mobile phase	50% acetonitrile: 50%, 0.1% formic acid (pH: 3.2)	ESI	Positive ESI
			Nitrogen (drying gas; 350 °C) at 100 L/H flow rate
	Flow rate: 0.2 mL/min		Cone gas: 100 L/H flow rate
	Injection volume: 5.0 μL		The voltage of extractor: 3.0 (V)
Eclipse plus-C18 column	50 mm long		The voltage of RF lens: 0.1 (V)
	2.1 mm i.d.		Capillary voltage: 4 KV
	3.5 μm particle size	Collision cell	Argon gas (collision gas) at 0.14 mL/min flow rate
	T: 22.0 ± 2.0 °C	Mode	MRM

**Table 2:** Composition of the prepared NLC formulations.

Formulation Code *	Lipid Phase				Aqueous Phase		
	SA	OA	GEF	AZT	P-188	TPGS	Water
Plain-NLC	450	150	0	0	120	7.5	14,272.5
GEF-NLC	450	150	15	0	120	7.5	14,257.5
AZT-NLC	450	150	0	15	120	7.5	14,257.5
GEF-AZT-NLC	450	150	15	15	120	7.5	14,242.5

* The amount was expressed in mg units. Plain-NLC (drug-free nanostructured lipid carrier), GEF-NLC (Gefitinib-loaded nanostructured lipid carrier), AZT-NLC (Azacitidine-loaded nanostructured lipid carrier), GEF-AZT-NLC (Azacitidine-Gefitinib-loaded nanostructured lipid carrier), stearic acid (stearic acid), OA (oleic acid), GEF (Gefitinib), AZT (Azacitidine), P-188 (poloxamer 188), TPGS (D-α-Tocopherol polyethylene glycol 1000 succinate).

**Table 3:** Physicochemical properties of prepared NLC formulations.

Measured Parameter *		Plain-NLC	GEF-NLC	AZT-NLC	GEF-AZT-NLC
Particle size (nm)		258.0 ± 11.2	272.1 ± 21.3	235.1 ± 11.8	268.9 ± 23.6
Polydispersity index		0.171 ± 0.01	0.129 ± 0.09	0.149 ± 0.05	0.204 ± 0.08
Zeta potential (mV)		−31.1 ± 3.3	−23.9 ± 1.2	−17.0 ± 2.0	−15.3 ± 1.2
Drug content (mg/mL)	GEF	---	1.01 ± 0.2	---	1.04 ± 0.3
	AZT	---	---	1.03 ± 0.5	0.98 ± 0.5
Entrapment efficiency (%)	GEF	---	97.2 ± 0.8	---	96.7 ± 0.6
	AZT	---	---	96.8 ± 1.1	98.3 ± 0.9

* Data are expressed as the mean ± SD, N = 3.

**Table 4:** IC_50_ of GEF, AZT, and GEF + AZT on cell viability of lung cancer cell line after 24 h.

Anticancer Agent	IC_50_ (μg/mL) *
GEF	11.14 ± 0.44
AZT	4.89 ± 0.22
GEF + AZT	4.31 ± 0.10

* Data are expressed as the mean ± SD, N = 3.

## Data Availability

Data will be made available on request.

## References

[1] Deng Y., Zhao P., Zhou L., Xiang D., Hu J., Liu Y., Ruan J., Ye X., Zheng Y., Yao J. (2020). Epidemiological trends of tracheal, bronchus, and lung cancer at the global, regional, and national levels: A population-based study. J. Hematol. Oncol..

[2] Shanker M., Willcutts D., Roth J.A., Ramesh R. (2010). Drug resistance in lung cancer. Lung Cancer Targets Ther..

[3] Huang J., Deng G., Wang S., Zhao T., Chen Q., Yang Y., Yang Y., Zhang J., Nan Y., Liu Z. (2023). A NIR-II Photoactivatable “ROS Bomb” with High-Density Cu_2_O-Supported MoS_2_ Nanoflowers for Anticancer Therapy. Adv. Sci..

[4] Yang Y., Huang J., Liu M., Qiu Y., Chen Q., Zhao T., Xiao Z., Yang Y., Jiang Y., Huang Q. (2023). Emerging Sonodynamic Therapy-Based Nanomedicines for Cancer Immunotherapy. Adv. Sci..

[5] Gong J., Shi T., Liu J., Pei Z., Liu J., Ren X., Li F., Qiu F. (2023). Dual-drug codelivery nanosystems: An emerging approach for overcoming cancer multidrug resistance. Biomed. Pharmacother..

[6] Hu Y., Zhang J., Hu H., Xu S., Xu L., Chen E. (2020). Gefitinib encapsulation based on nano-liposomes for enhancing the curative effect of lung cancer. Cell Cycle.

[7] Javadi S., Zhiani M., Mousavi M.A., Fathi M. (2020). Crosstalk between Epidermal Growth Factor Receptors (EGFR) and integrins in resistance to EGFR tyrosine kinase inhibitors (TKIs) in solid tumors. Eur. J. Cell Biol..

[8] Sherif A.Y., Harisa G.I., Alanazi F.K. (2023). The Chimera of TPGS and Nanoscale Lipid Carriers as Lymphatic Drug Delivery Vehicles to Fight Metastatic Cancers. Curr. Drug Deliv..

[9] Sherif Y.A., Harisa I.G., Alanazi K.F. (2023). SLN Mediate Active Delivery of Gefitinib into A549 Cell Line: Optimization, Biosafety, and Cytotoxicity Studies. Drug Deliv. Lett..

[10] Wang H., Huang Y. (2020). Combination therapy based on nano codelivery for overcoming cancer drug resistance. Med. Drug Discov..

[11] Müller A.M., Florek M. (2014). 5-Azacytidine/5-Azacitidine. Small Molecules in Oncology.

[12] Vendetti F.P., Topper M., Huang P., Dobromilskaya I., Easwaran H., Wrangle J., Baylin S.B., Poirier J.T., Rudin C.M. (2015). Evaluation of azacitidine and entinostat as sensitization agents to cytotoxic chemotherapy in preclinical models of non-small cell lung cancer. Oncotarget.

[13] Cheng H., Zou Y., Shah C.D., Fan N., Bhagat T.D., Gucalp R., Kim M., Verma A., Piperdi B., Spivack S.D. (2021). First-in-human study of inhaled Azacitidine in patients with advanced non-small cell lung cancer. Lung Cancer.

[14] Sharma A., Shambhwani D., Pandey S., Singh J., Lalhlenmawia H., Kumarasamy M., Singh S.K., Chellappan D.K., Gupta G., Prasher P. (2022). Advances in lung cancer treatment using nanomedicines. ACS Omega.

[15] Ejigah V., Owoseni O., Bataille-Backer P., Ogundipe O.D., Fisusi F.A., Adesina S.K. (2022). Approaches to improve macromolecule and nanoparticle accumulation in the tumor microenvironment by the enhanced permeability and retention effect. Polymers.

[16] Huang Q., Yang Y., Zhu Y., Chen Q., Zhao T., Xiao Z., Wang M., Song X., Jiang Y., Yang Y. (2023). Oral Metal-Free Melanin Nanozymes for Natural and Durable Targeted Treatment of Inflammatory Bowel Disease (IBD). Small.

[17] Song X., Huang Q., Yang Y., Ma L., Liu W., Ou C., Chen Q., Zhao T., Xiao Z., Wang M. (2023). Efficient Therapy of Inflammatory Bowel Disease (IBD) with Highly Specific and Durable Targeted Ta2C Modified with Chondroitin Sulfate (TACS). Adv. Mater..

[18] Huang Q., Liu Z., Yang Y., Yang Y., Huang T., Hong Y., Zhang J., Chen Q., Zhao T., Xiao Z. (2023). Selenium Nanodots (SENDs) as Antioxidants and Antioxidant-Prodrugs to Rescue Islet β Cells in Type 2 Diabetes Mellitus by Restoring Mitophagy and Alleviating Endoplasmic Reticulum Stress. Adv. Sci..

[19] Harisa G.I., Sherif A.Y., Alanazi F.K. (2023). Hybrid Lymphatic Drug Delivery Vehicles as a New Avenue for Targeted Therapy: Lymphatic Trafficking, Applications, Challenges, and Future Horizons. J. Membr. Biol..

[20] Wang L., Liu G., Hu Y., Gou S., He T., Feng Q., Cai K. (2022). Doxorubicin-loaded polypyrrole nanovesicles for suppressing tumor metastasis through combining photothermotherapy and lymphatic system-targeted chemotherapy. Nanoscale.

[21] Munir R., Lisec J., Swinnen J.V., Zaidi N. (2019). Lipid metabolism in cancer cells under metabolic stress. Br. J. Cancer.

[22] Butler L.M., Perone Y., Dehairs J., Lupien L.E., de Laat V., Talebi A., Loda M., Kinlaw W.B., Swinnen J.V. (2020). Lipids and cancer: Emerging roles in pathogenesis, diagnosis and therapeutic intervention. Adv. Drug Deliv. Rev..

[23] Sherif A.Y., Harisa G.I., Alanazi F.K., Nasr F.A., Alqahtani A.S. (2022). Engineered Nanoscale Lipid-Based Formulation as Potential Enhancer of Gefitinib Lymphatic Delivery: Cytotoxicity and Apoptotic Studies Against the A549 Cell Line. AAPS PharmSciTech.

[24] Fan W., Yu Z., Peng H., He H., Lu Y., Qi J., Dong X., Zhao W., Wu W. (2020). Effect of particle size on the pharmacokinetics and biodistribution of parenteral nanoemulsions. Int. J. Pharm..

[25] Son G.H., Na Y.G., Huh H.W., Wang M., Kim M.K., Han M.G., Byeon J.J., Lee H.K., Cho C.W. (2019). Systemic design and evaluation of ticagrelor-loaded nanostructured lipid carriers for enhancing bioavailability and antiplatelet activity. Pharmaceutics.

[26] Sherif A.Y., Harisa G.I., Alanazi F.K., Nasr F.A., Alqahtani A.S. (2022). PEGylated SLN as a promising approach for lymphatic delivery of gefitinib to lung cancer. Int. J. Nanomed..

[27] Hasan M.K., Enomoto K., Kikuchi M., Narumi A., Takahashi S., Kawaguchi S. (2023). Dispersion of submicron-sized SiO_2_/Al_2_O_3_-coated TiO_2_ particles and efficient encapsulation via the emulsion copolymerization of methacrylates using a thermoresponsive polymerizable nonionic surfactant. Polym. J..

[28] Harisa G.I., Sherif A.Y., Alanazi F.K., Ali E.A., Omran G.A., Nasr F.A., Attia S.M., Alqahtani A.S. (2023). TPGS decorated NLC shift gefitinib from portal absorption into lymphatic delivery: Intracellular trafficking, biodistribution and bioavailability studies. Colloids Surf. B Biointerfaces.

[29] Kesharwani P., Md S., Alhakamy N.A., Hosny K.M., Haque A. (2021). QbD enabled azacitidine loaded liposomal nanoformulation and its in vitro evaluation. Polymers.

[30] Rohilla S., Awasthi R., Mehta M., Chellappan D.K., Gupta G., Gulati M., Singh S.K., Anand K., Oliver B.G., Dua K. (2022). Preparation and Evaluation of Gefitinib Containing Nanoliposomal Formulation for Lung Cancer Therapy. BioNanoScience.

[31] Moura R.B.P., Andrade L.M., Alonso L., Alonso A., Marreto R.N., Taveira S.F. (2022). Combination of lipid nanoparticles and iontophoresis for enhanced lopinavir skin permeation: Impact of electric current on lipid dynamics. Eur. J. Pharm. Sci..

[32] Owens K., Argon S., Yu J., Yang X., Wu F., Lee S.C., Sun W.J., Ramamoorthy A., Zhang L., Ragueneau-Majlessi I. (2021). Exploring the Relationship of Drug BCS Classification, Food Effect, and Gastric pH-Dependent Drug Interactions. AAPS J..

[33] Perera G., Zipser M., Bonengel S., Salvenmoser W., Bernkop-Schnürch A. (2015). Development of phosphorylated nanoparticles as zeta potential inverting systems. Eur. J. Pharm. Biopharm..

[34] Fernandes R.S., Silva J.O., Monteiro L.O., Leite E.A., Cassali G.D., Rubello D., Cardoso V.N., Ferreira L.A., Oliveira M.C., de Barros A.L. (2016). Doxorubicin-loaded nanocarriers: A comparative study of liposome and nanostructured lipid carrier as alternatives for cancer therapy. Biomed. Pharmacother..

[35] Ghate V.M., Kodoth A.K., Shah A., Vishalakshi B., Lewis S.A. (2019). Colloidal nanostructured lipid carriers of pentoxifylline produced by microwave irradiation ameliorates imiquimod-induced psoriasis in mice. Colloids Surf. B Biointerfaces.

[36] Ebrahimi S., Farhadian N., Karimi M., Ebrahimi M. (2020). Enhanced bactericidal effect of ceftriaxone drug encapsulated in nanostructured lipid carrier against gram-negative Escherichia coli bacteria: Drug formulation, optimization, and cell culture study. Antimicrob. Resist. Infect. Control.

[37] Yang X., Zhao L., Almasy L., Garamus V.M., Zou A., Willumeit R., Fan S. (2013). Preparation and characterization of 4-dedimethylamino sancycline (CMT-3) loaded nanostructured lipid carrier (CMT-3/NLC) formulations. Int. J. Pharm..

[38] Argemí A., Vega A., Subra-Paternault P., Saurina J. (2009). Characterization of azacytidine/poly (l-lactic) acid particles prepared by supercritical antisolvent precipitation. J. Pharm. Biomed. Anal..

[39] Ribeiro M.D.M., Arellano D.B., Grosso C.R.F. (2012). The effect of adding oleic acid in the production of stearic acid lipid microparticles with a hydrophilic core by a spray-cooling process. Food Res. Int..

[40] Nayek S., Raghavendra N., Kumar B.S. (2021). Development of novel S PC-3 gefitinib lipid nanoparticles for effective drug delivery in breast cancer. Tissue distribution studies and cell cytotoxicity analysis. J. Drug Deliv. Sci. Technol..

[41] Alshetaili A.S. (2021). Gefitinib loaded PLGA and chitosan coated PLGA nanoparticles with magnified cytotoxicity against A549 lung cancer cell lines. Saudi J. Biol. Sci..

[42] Galvao J.G., Trindade G.G., Santos A.J., Santos R.L., Chaves Filho A.B., Lira A.A., Miyamoto S., Nunes R.S. (2016). Effect of Ouratea sp. butter in the crystallinity of solid lipids used in nanostructured lipid carriers (NLCs). J. Therm. Anal. Calorim..

[43] Lin Y., Yin W., Li Y., Liu G. (2022). Influence of different solid lipids on the properties of a novel nanostructured lipid carrier containing Antarctic krill oil. Int. J. Food Sci. Technol..

[44] Jansook P., Fülöp Z., Ritthidej G.C. (2019). Amphotericin B loaded solid lipid nanoparticles (SLNs) and nanostructured lipid carrier (NLCs): Physicochemical and solid-solution state characterizations. Drug Dev. Ind. Pharm..

[45] Pandey S.S., Patel M.A., Desai D.T., Patel H.P., Gupta A.R., Joshi S.V., Shah D.O., Maulvi F.A. (2020). Bioavailability enhancement of repaglinide from transdermally applied nanostructured lipid carrier gel: Optimization, in vitro and in vivo studies. J. Drug Deliv. Sci. Technol..

[46] Holder J.E., Ferguson C., Oliveira E., Lodeiro C., Trim C.M., Byrne L.J., Bertolo E., Wilson C.M. (2023). The use of nanoparticles for targeted drug delivery in non-small cell lung cancer. Front. Oncol..

[47] Almoustafa H.A., Alshawsh M.A., Al-Suede FS R., Alshehade S.A., Abdul Majid AM S., Chik Z. (2023). The Chemotherapeutic Efficacy of Hyaluronic Acid Coated Polymeric Nanoparticles against Breast Cancer Metastasis in Female NCr-Nu/Nu Nude Mice. Polymers.

[48] Makeen H.A., Mohan S., Al-Kasim M.A., Attafi I.M., Ahmed R.A., Syed N.K., Sultan M.H., Al-Bratty M., Alhazmi H.A., Safhi M.M. (2020). Gefitinib loaded nanostructured lipid carriers: Characterization, evaluation and anti-human colon cancer activity in vitro. Drug Deliv..

[49] De K. (2021). Decapeptide modified doxorubicin loaded solid lipid nanoparticles as targeted drug delivery system against prostate cancer. Langmuir.

[50] Kashyap K., Handa M., Shukla R. (2020). Azacitidine loaded PLGA nanoparticles and their dual release mechanism. Curr. Nanomed. (Former. Recent Pat. Nanomed.).

[51] Zhou W., Jia Y., Liu Y., Chen Y., Zhao P. (2022). Tumor Microenvironment-Based Stimuli-Responsive Nanoparticles for Controlled Release of Drugs in Cancer Therapy. Pharmaceutics.

[52] Zhang C., Peng F., Liu W., Wan J., Wan C., Xu H., Lam C.W., Yang X. (2014). Nanostructured lipid carriers as a novel oral delivery system for triptolide: Induced changes in pharmacokinetics profile associated with reduced toxicity in male rats. Int. J. Nanomed..

[53] Zhang J., Li S., An F.F., Liu J., Jin S., Zhang J.C., Wang P.C., Zhang X., Lee C.S., Liang X.J. (2015). Self-carried curcumin nanoparticles for in vitro and in vivo cancer therapy with real-time monitoring of drug release. Nanoscale.

[54] Salvi V.R., Pawar P. (2019). Nanostructured lipid carriers (NLC) system: A novel drug targeting carrier. J. Drug Deliv. Sci. Technol..

[55] Wang J., Wang F., Li X., Zhou Y., Wang H., Zhang Y. (2019). Uniform carboxymethyl chitosan-enveloped Pluronic F68/poly (lactic-co-glycolic acid) nano-vehicles for facilitated oral delivery of gefitinib, a poorly soluble antitumor compound. Colloids Surf. B Biointerfaces.

[56] Huang Y., Dai Y., Wen C., He S., Shi J., Zhao D., Wu L., Zhou H. (2020). circSETD3 contributes to acquired resistance to gefitinib in non-small-cell lung cancer by targeting the miR-520h/ABCG2 pathway. Mol. Ther. Nucleic Acids.

[57] Sherif A.Y., Harisa G.I., Shahba A.A., Alanazi F.K., Qamar W. (2023). Optimization of Gefitinib-Loaded Nanostructured Lipid Carrier as a Biomedical Tool in the Treatment of Metastatic Lung Cancer. Molecules.

[58] Abdolahpour S., Mahdieh N., Jamali Z., Akbarzadeh A., Toliyat T., Paknejad M. (2017). Development of doxorubicin-loaded nanostructured lipid carriers: Preparation, characterization, and in vitro evaluation on MCF-7 cell line. BioNanoScience.

[59] Zhang X.-G., Miao J., Dai Y.-Q., Du Y.-Z., Yuan H., Hu F.-Q. (2008). Reversal activity of nanostructured lipid carriers loading cytotoxic drug in multi-drug resistant cancer cells. Int. J. Pharm..

[60] Shah P., Chavda K., Vyas B., Patel S. (2021). Formulation development of linagliptin solid lipid nanoparticles for oral bioavailability enhancement: Role of P-gp inhibition. Drug Deliv. Transl. Res..

[61] Tanaudommongkon I., Tanaudommongkon A., Prathipati P., Nguyen J.T., Keller E.T., Dong X. (2020). Curcumin nanoparticles and their cytotoxicity in docetaxel-resistant castration-resistant prostate cancer cells. Biomedicines.

[62] Hiremath C.G., Heggnnavar G.B., Kariduraganavar M.Y., Hiremath M.B. (2019). Co-delivery of paclitaxel and curcumin to foliate positive cancer cells using Pluronic-coated iron oxide nanoparticles. Prog. Biomater..

[63] Tran N., Mulet X., Hawley A.M., Hinton T.M., Mudie S.T., Muir B.W., Giakoumatos E.C., Waddington L.J., Kirby N.M., Drummond C.J. (2015). Nanostructure and cytotoxicity of self-assembled monoolein–capric acid lyotropic liquid crystalline nanoparticles. RSC Adv..

[64] Wu K.W., Sweeney C., Dudhipala N., Lakhani P., Chaurasiya N.D., Tekwani B.L., Majumdar S. (2021). Primaquine loaded solid lipid nanoparticles (SLN), nanostructured lipid carriers (NLC), and nanoemulsion (NE): Effect of lipid matrix and surfactant on drug entrapment, in vitro release, and ex vivo hemolysis. AAPS PharmSciTech.

